# Arthroscopic Lysis of Adhesions for Treatment of Post-Traumatic Knee Arthrofibrosis: A Prospective Study

**DOI:** 10.7759/cureus.33275

**Published:** 2023-01-02

**Authors:** Karim M Abdelghafour, Ahmed S Elkalyoby, Mohammad H Amer, M. K Abdelaziz

**Affiliations:** 1 Trauma and Orthopaedics, Cairo University, Cairo, EGY; 2 Trauma and Orthopaedics, East and North Hertfordshire NHS (National Health Service) Trust Lister Hospital, Stevenage, GBR; 3 Trauma and Orthopaedics, Royal Sussex County Hospital, University Hospitals of Sussex, Brighton, GBR

**Keywords:** lysholm score, adhesions, range of motion, stiffness, arthroscopic lysis, knee arthrofibrosis

## Abstract

Background: Post-traumatic knee arthrofibrosis can have a significant effect on patients’ function and the management can be challenging with a variety of options ranging from manipulation under anaesthesia (MUA) to more invasive procedures such as quadricepsplasty. The aim of the study is to evaluate the improvement of both knee range of motion (ROM) and functional outcomes after arthroscopic lysis of adhesions (ALA) for post-traumatic knee arthrofibrosis.

Materials and Methods: A prospective study of 30 patients with post-traumatic knee arthrofibrosis was managed by arthroscopic arthrolysis. Aggressive rehabilitation protocol was initiated on the first day postoperatively. Comprehensive clinical follow-up evaluations including the ROM assessment and the Lysholm score were done for all patients.

Results: The mean age was 36.17 years (±9.51). The mean follow-up time was 6.7 months (six to nine months). The ROM improved from 75° (±10.91°) preoperatively to 119.83° (± 10.38°) at the final follow-up (P < 0.001). Additionally, the Lysholm score increased from 56.90(±2.64) preoperatively to 85.27(±3.46) (P < 0.001). The ultimate final ROM and functional outcomes for all patients were satisfactory.

Conclusion: ALA for knee arthrofibrosis significantly improves the knee ROM and functional outcomes and can be a successful alternative to open quadricepsplasty.

## Introduction

Post-traumatic knee arthrofibrosis remains a complex complication after knee surgery or trauma. It is a well-recognized phenomenon and has been reported in as many as 11% of cases in developed countries [1] and may be much higher in developing countries. A large percentage of these cases present with fibrosis inside as well as outside the knee [2,3].

Prolonged knee immobilisation and inadequate rehabilitation are the main contributors to post-traumatic knee arthrofibrosis. It is a progressive process that comprises excessive fibrous scar tissue proliferation, periarticular soft tissue retraction, and bone impingement that can range from localized areas to generalized involvement, which finally leads to the development of extensive extra-articular adhesions of the quadriceps [4]. A variety of surgical techniques have been developed, including traditional techniques such as the Thompson quadricepsplasty and Judet quadricepsplasty and their variations [5,6]. However, these procedures are associated with high rates of complications, such as postoperative skin sloughing and extension lag. Accordingly, less invasive procedures were implemented, including manipulation under anaesthesia (MUA), intra-articular adhesiolysis, extra-articular mini-incision release, and ‘‘multiple Z" lengthening of the quadriceps tendon besides an early aggressive rehabilitation protocol. These minimally invasive techniques have a favourable prognosis regarding morbidity and outcome [2,6].

Whilst achieving an effective operative plan for management is imperative, other treatment variables such as the time from initial injury to the intervention, and the effectiveness and compliance of the patient to the rehabilitation protocol remain debatable. Knowledge of these management options and potential complications is beneficial in the management of post-traumatic knee stiffness [7]. The purpose of this study is to assess the effectiveness of surgical arthroscopic lysis of knee adhesions (ALA) for post-traumatic arthrofibrosis regarding the improvement of both the functional range of motion (ROM) and the functional knee score (Lysholm score). 

## Materials and methods

Thirty patients were selected prospectively for arthroscopic lysis of adhesions (ALA) and outcome measurement following post-traumatic arthrofibrosis between August 2019 and June 2021 in Cairo University Hospitals, Cairo, Egypt. This study was approved in February 2019 by the Post Graduate Affairs and Research Office, Faculty of Medicine, Cairo University, Cairo, Egypt (approval number: 2019/00112).

Inclusion Criteria were patients with limitation of flexion to less than 120 degrees after surgically treated ipsilateral peri-articular or diaphyseal fractures not responding to physiotherapy for more than three months. Skeletally immature individuals, non-traumatic cases of knee stiffness (synovial lesions such as rheumatoid arthritis, pigmented villonodular synovitis, or post-total knee arthroplasty cases), post knee ligamentous reconstruction, the presence of severe knee osteoarthritis [8] were excluded. Patients were evaluated clinically by history taking for the trauma or surgery and the duration until the arthroscopic lysis. All patients were examined for passive and active ROM, patellar mobility, suppleness of the patellar tendon, stability of the knee, and gait. Medical history with special attention to co-morbidities such as diabetes, hypertension, renal failure, and pre-injury level of activity. Lysholm knee score was done for all patients. Plain radiographs (anteroposterior and lateral) views were routine in all cases for assessment of union of fractures. A lateral view helped assess for the detection of Patella Baja/infra or the presence of patellofemoral arthrosis.

Statistical method

IBM SPSS Statistics for Windows, Version 28.0 ( Released 2021; IBM Corp., Armonk, New York, United States) was used for data coding and entry. Mean and standard deviation were used to summarise the data. For comparison of serial measurements within the same patient, paired t-test and repeated measures ANOVA were used [9]. Correlations between quantitative variables were done using the Pearson correlation coefficient [10]. P-values less than 0.05 were considered statistically significant.

Surgical technique

**Figure 1:**
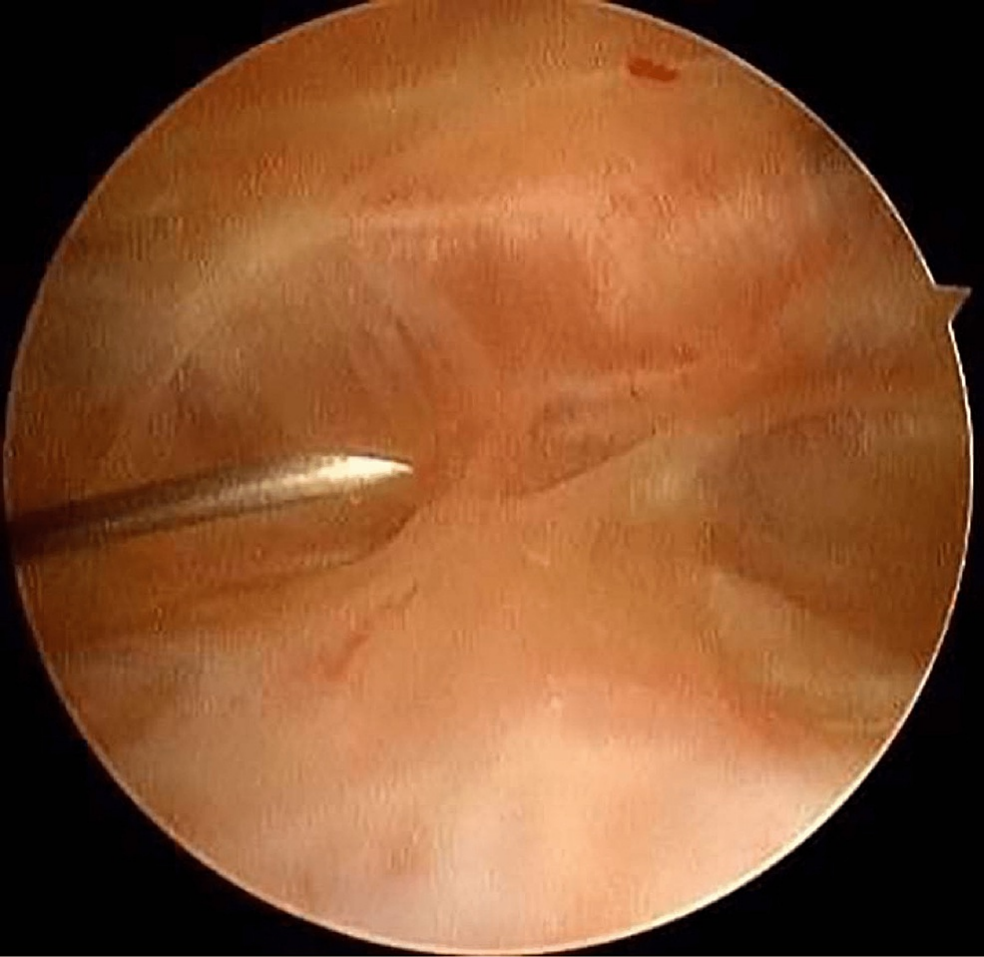
Arthroscopic view of the suprapatellar region of the right knee: suprapatellar adhesions and making the accessory suprapatellar portal using a needle.

**Figure 2:**
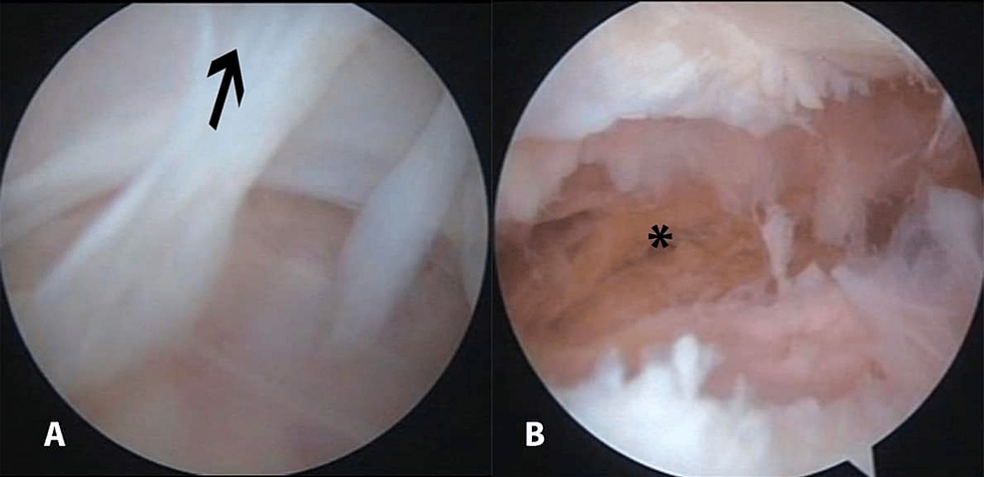
Arthroscopic Image from the suprapatellar pouch of the right knee: (A) Adhesions filling the suprapatellar pouch (arrow); (B) Adhesions after release with the arthroscopic shaver (asterisk).

**Figure 3:**
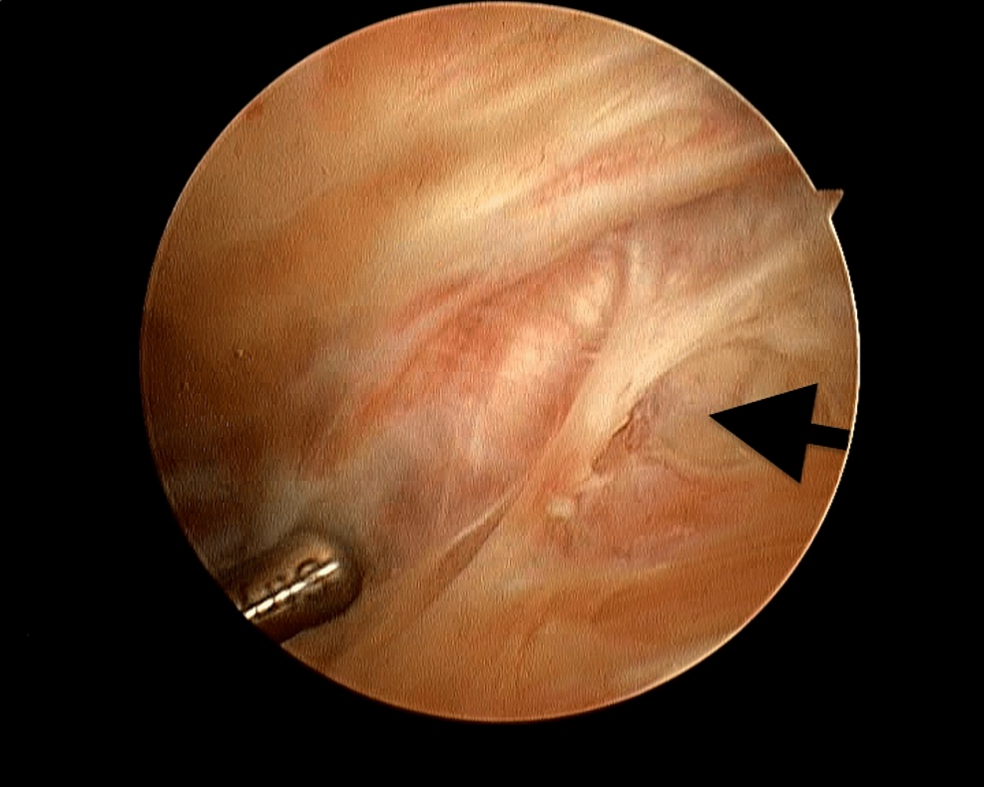
Arthroscopic view of the lateral gutter of the right knee: release of adhesions from the lateral gutter (arrow)

All patients were anaesthetized by regional spinal anaesthesia and all surgeries were performed in the supine position on the operating table. Routine examination under anaesthesia (EUA) for all patients was done, the ROM was examined using a goniometer and also patellar mobility concerning medial-lateral glide, superior-inferior glide, and patellar tilt were examined. A tourniquet was applied to the thigh with the knee in the maximum possible flexion position to minimise capturing of the quadriceps femoris muscle. The procedure was done using the three-portal technique using the two standard anterolateral and anteromedial portals in addition to the accessory superolateral portal. Tissue adhesions were identified and debrided with an arthroscopic shaver. The anterolateral portal was inserted first followed by the superolateral portal 2cm superior to the upper border of the patella using the out-in technique whilst the knee was in full extension (Figure 1). As the whole knee spaces are obliterated by the adhesions, the order of the operative steps is key to the success of the procedure overall. The adhesions were released from the whole suprapatellar pouch through the superolateral portal starting from the most proximal part and down to the trochlea (Figures 2A, 2B) then the lateral gutter was released (Figure 3).

**Figure 4:**
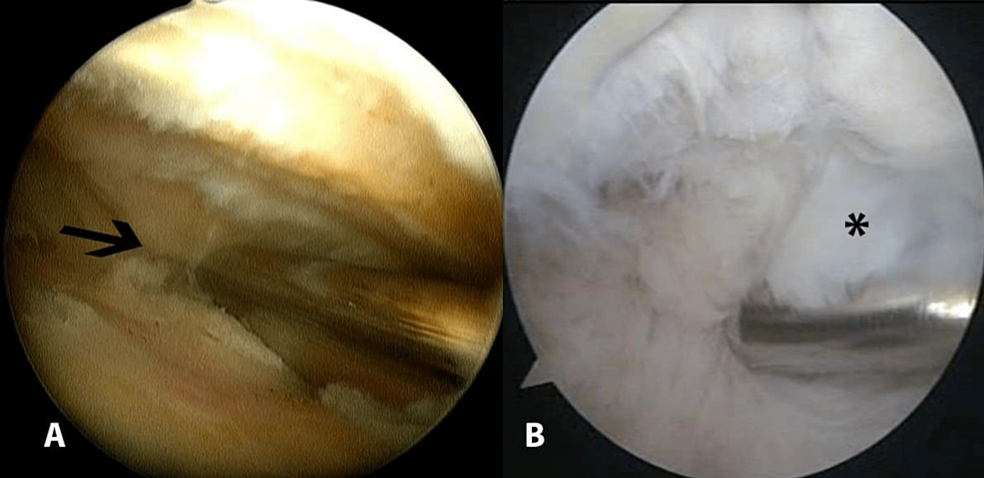
Release of adhesions from (A) medial gutter (arrow) and (B) intercondylar notch (asterisk) of the left knee.

The anteromedial portal was then developed and, through it, the medial gutter and retropatellar area were released followed by the release of the intercondylar notch and anterior interval adhesions (Figures 4A, 4B). Articular cartilage could be seen after the excision of the obliterating fibrous tissue. Anterior cruciate ligament (ACL) and posterior cruciate ligament (PCL) femoral insertion could be seen after adhesions between the infrapatellar fat pad and ligamentum mucosum were removed. Release between the ACL and PCL from the adhesions was performed. The whole articular cartilage of the knee joint was carefully examined and chondroplasty was performed, when necessary. The fibrous tissue attached to the chondral surface for a prolonged duration can cause irreversible chondral damage. Debriding the chondral tissue completely of the fibrotic bands is therefore necessary. Following the release of adhesions, manipulation of the knee was done to assess the gain of the ROM obtained by this arthroscopic release.

Postoperative protocol 

For 24 hours postoperatively, an elastic bandage was wrapped from the toes to the thigh and this was followed by regular dressings and ice packs to reduce oedema. Isometric quadriceps exercise was encouraged on the first postoperative day. Immediate postoperative X-rays were obtained to check for any possible complications as iatrogenic fractures from the manipulation and neurovascular status were examined. Appropriate postoperative epidural analgesia was essential to maintain the ROM. An intensive course of physiotherapy was commenced postoperatively and continuous passive motion (CPM) was applied for use three times a day. Immediate active-assisted followed by an active ROM exercise program was started and the muscle strengthening exercise program for quadriceps and hamstrings. Patients commenced early assisted weight-bearing and were encouraged to fully weight-bear as tolerated.

Postoperative evaluation

ROM was assessed immediately post surgery and after six months of physiotherapy. Lysholm score was calculated preoperatively and after six months.

## Results

This study included 30 patients with post-traumatic knee arthrofibrosis who met the inclusion criteria treated with ALA. All cases were followed up for at least six months with a mean follow-up period of 6.7 months. The mean age was 36.17 (22-52) years ±9.51. There were 22 males (73.3%) and eight females (26.7%). Twelve patients were smokers (40%), while 18 patients were non-smokers (60%). Eight patients (26.7%) had known co-morbidities, while 22 patients (73.3%) did not have co-morbidities. Five patients had diabetes, two had bronchial asthma, and one was hepatitis C virus (HCV) positive. Fourteen patients (46.7%) had the left side affected and 16 patients (53.3%) had the right side affected.

All patients had previously surgically-managed traumatic injuries: 12 patients (40%) experienced distal femoral fractures (including supracondylar, intercondylar, or medial femoral condyle) that were surgically managed with plate fixation, four patients (13.3%) had antegrade femoral nail insertion, while four patients (13.3%) had retrograde femoral nail insertion, seven patients (23.3%) had tibial plateau fractures, and three (10%) had sustained patellar fractures. The mean duration between the surgery and the arthrolysis was 8.47 months ± 4.58 with a range between 3-21 months. 

Preoperative mean arc of motion was 75° ±10.91 (range: 55° - 90°). Immediate postoperative mean arc was 116.33° ± 8.40 (ranging between 95° and 130°). While the mean after six months was 119.83° ± 10.38 (range: 90° - 130°) and that was a statistically significant improvement in the ROM (Table 1).

**Table 1:** Comparison between preoperative, immediate postoperative, and six months postoperative ROM and between preoperative and postoperative Lysholm score. ROM: range of motion

	Mean	SD	Minimum	Maximum	P-value
Preoperative ROM	75.00	10.91	55.00	90.00	----
Immediate postoperative ROM	116.33	8.40	95.00	130.00	<0.001
Six months postoperative ROM	119.83	10.38	90.00	130.00	<0.001
Preoperative Lysholm score	56.90	2.64	53.00	61.00	<0.001
Postoperative Lysholm score	85.27	3.46	79.00	90.00	

**Figure 5:**
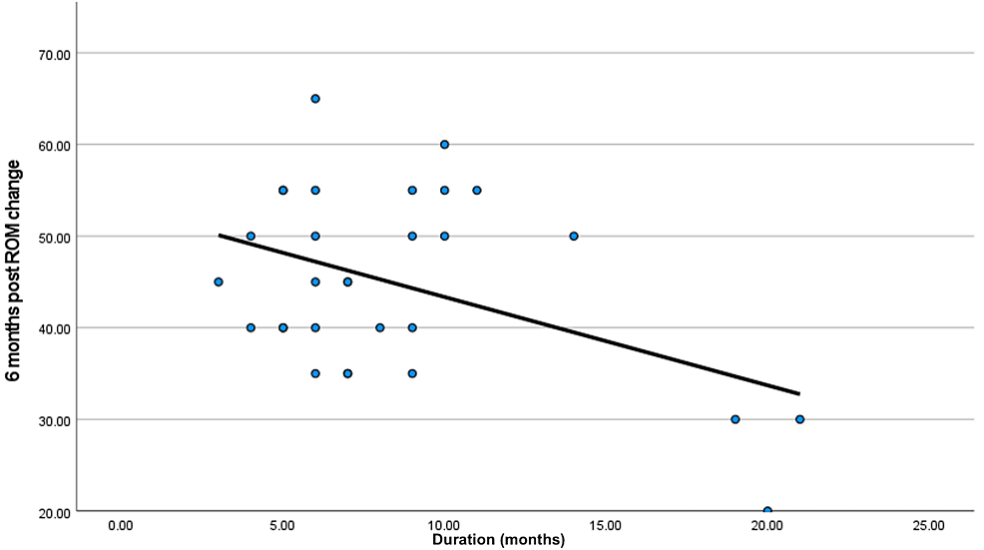
Correlation between improvement of range of motion and the duration between the surgery and the arthrolysis in months. ROM: range of motion

There was a significant inverse correlation between the interval between the previous surgery and the improvement in six months’ postoperative ROM (P-value 0.016), where any increase in the duration of stiffness (the interval between the previous surgery and the arthrolysis) is associated with poorer outcomes and less desirable improvement of the postoperative ROM (Table 2, Figure 5).

**Table 2:** Correlation between improvement of range of motion and the duration between the surgery and the arthrolysis. ROM: range of motion

		ROM change post six months
Duration	R	-0.436-
	P-value	0.016
	N	30

There was one case complicated with deep venous thrombosis, Four patients (13.3%) had decreased ROM around two to three weeks postoperatively in relation to the ROM in the early postoperative period. This phenomenon is referred to as ‘‘transient rebound’’ [2] and slow but steady progressive improvement in ROM continued after six to eight weeks postoperatively. There were no revision surgeries needed.

## Discussion

Management of post-traumatic knee joint stiffness is among the most difficult challenges in orthopaedics. Patients' quality of life and ability to work can be negatively impacted by post-traumatic knee arthrofibrosis. Minor loss of motion in terminal extension can significantly alter knee function and increases a patient’s risk of patellofemoral arthritis [2,11,12]. In the past, open surgical methods have been thought to be the most beneficial in the management of knee arthrofibrosis; however, Thompson quadricepsplasty, Judet quadricepsplasty, and their modifications, which are more invasive procedures aiming to provide more access to the knee joint, are associated with significant morbidity [5,6,13].

ALA is now a standardised technique that can be considered from three months after the injury event, and sometimes earlier. In fact, it should be suggested early on if the joint ROM is no longer improving assuming fractures have healed [14].

To evaluate the role of arthroscopic arthrolysis in the management of post-traumatic knee arthrofibrosis, 30 patients diagnosed with post-traumatic knee arthrofibrosis were managed with arthroscopic arthrolysis. We compared our results with those from similar studies in the literature. There were four studies that analysed the results of ALA in cases of post-traumatic knee arthrofibrosis and others analyse the outcomes of using the arthroscopic technique in comparison with conservative measures and open techniques.

Liu Z et al. performed a retrospective study of 25 patients with post-traumatic knee arthrofibrosis who had all-arthroscopic debridement with an evaluation of the improvement of knee ROM and Lysholm score [15]. Gittings et al. performed a retrospective study of 14 patients who underwent arthroscopic lysis for knee stiffness after intra-articular fractures and these patients were evaluated for the improvement of knee ROM both in the immediate postoperative and final follow-up period [16]. Liu S et al. performed a comparative study of arthroscopic lysis versus conservative management and minimally invasive quadricepsplasty. The study included 112 patients with post-traumatic knee stiffness with 24 of them treated arthroscopically. Clinical data were studied retrospectively and the differences in ROM and Hospital for Special Surgery (HSS) score of the knee before and after the treatment were also analyzed statistically [17]. Liu KM et al. completed a retrospective study where 20 consecutive patients managed with arthroscopic lysis of the intra-articular arthrofibrosis were evaluated for ROM and HSS knee score [2].

The majority of the cases of post-traumatic knee arthrofibrosis result from trauma or fractures around the knee. Liu Z et al. identified 19 out of 25 patients (76%) where the original trauma was around the knee with the majority of them being proximal tibial fractures [15]. In the study by Gittings et al., all 14 cases were a result of trauma around the knee (10 cases of tibial plateau fractures, three cases of patellar fractures and one case of distal femoral fracture) [16]. Liu S et al. also showed that all 24 cases included resulted from trauma around the knee [17]. In the study performed by Liu KM et al., 12 cases out of 20 cases (60%) resulted from trauma around the knee [2].

Regarding the interval between the trauma and arthrolysis, in our study, the mean was 8.47 months ±4.58; the minimum duration was three months and the maximum was 21 months. In the study performed by Liu S et al., the mean duration of stiffness was seven months (±8.4) [17], While in the study by Gittings et al., the average duration between the surgery and adhesiolysis was 244 days [16]. In the study by Liu KM et al. the mean duration of stiffness was 18 months (3-79) [2]. There was a significant inverse correlation between the interval between the previous surgery and the improvement in six months’ postoperative ROM (P-value 0.016), where any increase in the duration of stiffness is associated with poor outcomes and less desirable improvement of the postoperative ROM, which is coincident with Gittings et al. [16[ and Stiefel et al [12].

Regarding ROM in this study, the mean arc of motion preoperatively was 75° (±10.91°) improved in the immediate postoperative to a mean of 116.33° (±8.40°) and in the final six months follow-up period to a mean of 119.83° (± 10.38°) and that was a statistically significant improvement. In agreement with our study, Gittings et al. showed that ROM improved in patients with knee stiffness from 73° (±30°) to 127° (±8°) intraoperatively and final arc of motion had a mean of 104° (±29°) with P<0.01 [16]. Liu Z et al. showed improved ROM from 23.9° ± 7.5° preoperatively to 107.0° ± 7.5° on the first day postoperatively and 105.9° ± 6.5° at the final follow-up (P < 0.001) [15]. Liu S et al. showed improved ROM from 46° (± 27°) preoperatively to 117° (± 13.4°) during the final follow-up period [17]. Liu KM et al. showed significant improvement of the knee ROM [2]. On average, patients gained a ROM of 73° intra-operatively, 56° at discharge, and 77 °at the final follow-up visit. Flexion improved from a mean of 51° preoperation (range 15-80) to a mean of 100° (range 90-125) at discharge. All the improvements in ROM were statistically significant (p≤0.001).

The mean Lysholm score preoperatively was 56.90 (±2.64), which was improved to 85.27 (± 3.46) in the final follow-up with p-value<0.001. This data was comparable with Liu Z et al. where the mean Lysholm score by the follow-up period improved from 59.9 (± 5.2) to 89.7 (± 3.3) with P: < 0.001. Other studies such as Liu KM et al. and Liu S et al. used different knee scoring systems and their results were comparable with our study regarding the improvement of the functional outcome following the ALA.

The study has some limitations. First, the sample size is relatively small, which didn't allow better subgroup analysis between different injuries that led to arthrofibrosis and identifying other variables affecting the outcome that may have been underrepresented in this size. Second, the study included a single group of patients treated with the arthroscopic lysis without including a control group treated with simple MUA or more aggressive quadricepsplasty. A validation comparative study including the three cohorts of patients can add robust evidence to the current literature. Third, the study included only patients with post-traumatic arthrofibrosis without including patients who developed stiff knee after knee arthroplasty or after ligamentous reconstruction.

## Conclusions

ALA is a safe, simple, effective and feasible procedure in the management of arthrofibrosis with significant improvement of both knee ROM and functional outcomes. It is a minimally invasive technique with favourable results in comparison to open procedures such as quadricepsplasty with avoidance of the significant morbidity associated with those open procedures. Patient selection is crucial to surgical success. Early diagnosis of ROM loss and early arthroscopic lysis of adhesions may improve functional ROM in patients with a traumatic knee injury and motion loss. Patients with more severe, chronic motion loss may not benefit as much from arthroscopic procedures. The more prolonged the duration of stiffness the less improvement in both ROM and functional outcomes.
